# Transmission of *Mycobacterium tuberculosis* in a High School and School-Based Supervision of an Isoniazid-Rifapentine Regimen for Preventing Tuberculosis — Colorado, 2011–2012

**Published:** 2013-10-04

**Authors:** Carolyn Bargman, Randall Reves, Matt Parker, Robert Belknap, Juli Bettridge, Deborah T. Bedell, Maria E. Galvis, Christine S. Ho, John A. Jereb

**Affiliations:** Boulder County Public Health/Denver Metro TB Clinic; Denver Metro TB Clinic; Colorado Dept of Public Health and Environment; Div of Viral Hepatitis; Div of Tuberculosis Elimination, National Center for HIV/AIDS, Viral Hepatitis, STD, and TB Prevention, CDC

*Mycobacterium tuberculosis*, the bacterium that causes tuberculosis (TB), is spread from person to person by the airborne route. It can be transmitted extensively in congregate settings, making investigating exposures and treating infected contacts challenging. In December 2011, a student at a Colorado high school with 1,381 students and school personnel received a diagnosis of pulmonary TB disease. One of five household contacts had TB disease, and the other four had latent *M. tuberculosis* infection (LTBI). Screening of 1,249 school contacts (90%) found one person with pulmonary TB disease, who was fully treated, and 162 with LTBI, of whom 159 started an LTBI treatment regimen for preventing progression to TB disease and 153 completed a regimen. Only the index patient required inpatient care for TB, and TB caused no deaths. Use of short-course treatment regimens, either 12-dose weekly isoniazid and rifapentine directly observed at school or 4 months of self-supervised rifampin daily, facilitated treatment completion. State and county incident command structures led by county TB control authorities guided a response team from multiple jurisdictions. News media reports brought public scrutiny, but meetings with the community addressed the concerns and enhanced public participation. Two contacts of the index patient outside of the school had TB disease diagnosed after the school investigation. As of July 2013, no additional TB disease associated with in-school exposure had been found. An emergency plan for focusing widespread resources, an integral public communications strategy, and new, efficient interventions should be considered in other large TB contact investigations.

TB disease is confirmed by detection of *M. tuberculosis* by culture or nucleic acid amplification, or it can be diagnosed clinically from symptoms and chest radiography findings that are consistent with TB and resolve with treatment (1). In most instances, a clinical diagnosis includes positive results from an immunologic test for *M. tuberculosis* infection, either the tuberculin skin test (TST) or an interferon gamma release assay (IGRA) blood test (1,2). LTBI is diagnosed by positive TST or IGRA results, absence of TB disease symptoms, and a normal chest radiograph or a stable abnormal chest radiograph with tests of sputum negative for *M. tuberculosis* (1–3).

## Index Patient

In late December 2011, a student at a high school with 1,381 students and school personnel in Longmont, Colorado, was admitted to a hospital after 2 months of cough, fever, and night sweats. The student was U.S.-born, and the only TB risk (3) was living abroad at age 8–10 years in a country with a TB disease incidence 10 times greater than that for the United States. The chest radiograph showed a pulmonary cavity, and sputum-smear microscopy revealed acid-fast bacilli. Both findings are markers for potential contagiousness. The *M. tuberculosis* from sputum culture was susceptible to isoniazid, rifampin, ethambutol, and pyrazinamide, and treatment with the standard four-drug regimen was completed in September 2012.

## Contact Investigations

Persons who had spent the most time indoors with the index patient, as determined from interviews with the patient and later from school records, were tested for *M. tuberculosis* infection ahead of others (4). All five members of the household had positive IGRA results. One had culture-confirmed genito-urinary TB disease and a normal chest radiograph, with an *M. tuberculosis* genotype matching that of the index patient. A second person was initially treated for possible TB disease but after 2 months of a four-drug regimen was determined to have had LTBI (1,3). The other three had LTBI and took 4 months of daily rifampin for preventing TB disease (3).

The index patient’s six teachers and 13 students who shared at least two classes were tested by IGRA (Table). None had TB disease, but 10 (53%) of 19 had LTBI. Testing was then extended to 140 additional students who shared only one class with the patient. One received a diagnosis of TB disease initially, but the diagnosis was changed to LTBI after 2 months of four-drug treatment; 49 (35%) were diagnosed with LTBI.

The findings suggested *M. tuberculosis* transmission at the school; therefore, the investigation was extended to all students and school personnel enrolled or working during the fall 2011 semester. Because TB disease typically develops 6–18 months after initial *M. tuberculosis* infection (5), prompt diagnosis and treatment of LTBI were urgent. The rapid evaluation of approximately 1,000 additional contacts with IGRA was not feasible at the local laboratory; a combined strategy using IGRA and TST was adopted. Students and school personnel who had lived outside the United States, who had been vaccinated with bacille Calmette-Guérin (BCG), or who reported a positive TST result were tested with IGRA and all others with TST (2).

During 12 screening clinics held at the school from mid-February to mid-March, public health personnel interviewed and tested 1,053 contacts, and 37 more were screened in other settings, for a total of 1,090 (89.2%) of the 1,222 who were sought (Table). One had pulmonary TB disease diagnosed by chest radiography, but a negative sputum culture result, and was fully treated; 102 (8.3%) were diagnosed with LTBI. Combined with the earlier groups of school contacts, a total of 1,249 (90.4%) of 1,381 school contacts were evaluated: four who had previously been treated for LTBI were evaluated with a chest radiograph only, 435 were tested with IGRA, and 810 received TST.

## Treatment of Infected School Contacts

Contacts with LTBI were offered a choice between self-supervised daily isoniazid for 9 months or rifampin for 4 months until a rifapentine supply was secured in late February 2012. Then, once-weekly isoniazid and rifapentine for 12 weeks supervised at school by directly observed therapy (6) was recommended preferentially. Among the 90 contacts known to have been offered the latter regimen, 60 chose it, as well as five others for whom the options that were offered were not recorded. The workers supervising the doses used telephone calls, text messages, and home visits to encourage adherence and consulted daily with public health nurses about problems such as missed doses. Because rifampin and rifapentine can reduce the effectiveness of hormonal contraceptives, condoms were offered at the public health clinics.

Overall, 162 (13%) school contacts of the index patient had LTBI. This included the person who completed 2 months of four-drug treatment for TB disease before the diagnosis was changed to LTBI and whose treatment was regarded as sufficient (1,3). Of the remaining 161 contacts with LTBI, 159 (99%) started treatment, of whom 153 (96%) completed it. Treatment completion was similar by LTBI regimen: three of three (100%) for 9 months of isoniazid, 88 of 91 (97%) for 4 months of rifampin, and 61 of 65 (94%) for 12 doses of once-weekly isoniazid and rifapentine. One of the four not completing the latter regimen completed 9 months of isoniazid.

Of the three contacts who did not complete the rifampin regimen, two stopped for unknown reasons. The other had treatment interrupted because of an adverse event (rash). Then isoniazid was prescribed, but it was discontinued because of another adverse event (hepatitis). Of the four contacts not completing the isoniazid-rifapentine regimen, one stopped for unknown reasons after 6 doses. Three had their treatment interrupted because of adverse events. One had headache, nausea, and depression and completed 9 months of daily isoniazid. One had rash, dizziness, and blurred vision that recurred with daily isoniazid and declined further treatment. One had fever, aches, malaise, and interactions between rifapentine and other medications and declined further treatment.

## Response Capacity, Communications, and Community Relations

The surge in workload exceeded the capacity of the local health department. Colorado officials activated public health preparedness programs for the state and Denver and Boulder counties, which established an incident command structure led by the TB control authorities of Denver and Boulder counties. Eighty-one persons from seven city or county health departments and the state health department, two county medical reserve corps members, and representatives from two schools of nursing and one school district served at the in-school screening clinics, with 43 of these persons attending multiple sessions. Five registered nurses from four health departments, who were supported by one clerk, counseled the patients, administered the first directly observed doses of isoniazid-rifapentine and provided the monthly supplies of isoniazid or rifampin for daily self-supervised treatment. Two persons from CDC were reassigned from other state health department duties to supervise the weekly isoniazid-rifapentine regimen at the school, 1 month each consecutively, followed by two Denver Metro TB Clinic outreach workers who supervised doses at the school and then at homes after summer vacation began. The workload for the screening clinics was 885 person-hours and for the LTBI treatment was 890 person-hours, which included 560 person-hours for supervising isoniazid-rifapentine doses and tracing patients who missed doses. These measures of workload did not include the hours spent planning, keeping records, and communicating with the public or the hours spent investigating the earlier groups of contacts in the school and other settings.

In mid-January 2012, local news media began featuring the investigation. This was followed by Internet social media reporting, including perceptions that students were dying from TB, that illegal immigrants brought the TB, and that the school would be closed. Throughout the contact investigation, public health and school officials convened public meetings with school personnel, students, their families, and news reporters to address concerns and perceptions about TB. At follow-up meetings, the officials reported the progress of the investigation and showed evidence that transmission had ended. The importance of preventing future TB disease by completing LTBI treatment was stressed.

The index patient did not disclose 20 nonschool social contacts until December 2012, when one had developed pleural TB disease with negative culture results. Nine others were completely evaluated, and two had LTBI. The remaining 10 were either not found or not completely evaluated. One had left Colorado and was diagnosed with culture-confirmed TB disease in another jurisdiction in 2013. The genotypes for the *M. tuberculosis* isolates from that patient matched those from the index patient. As of July 2013, no additional TB disease in school contacts had been reported, and no additional *M. tuberculosis* isolates with this genotype had been found in Colorado.

### Editorial Note

Colorado is a low-incidence TB state, with 64 cases of TB disease reported in 2012 for a rate of less than 1.4 per 100,000 person-years, compared with 3.2 per 100,000 in 2012 for the United States overall. TB contact investigations, especially those in congregate settings, are intrinsically complex and labor-intensive (4), requiring mobilization of a large, flexible workforce for a prolonged response. This investigation at a high school demonstrated how an emergency response plan can gather widely dispersed expertise to one site. Augmenting the local health department with additional personnel expedited the evaluation of more than 1,200 contacts and the treatment of those who were infected. Transmission of *M. tuberculosis* at schools is unusual, but this investigation found that numerous contacts had been infected, particularly those who had shared classes with the index patient. The absence of TB disease in school contacts after the investigation indicates that the interventions were effective.

The Colorado experience with the weekly 12-dose isoniazid-rifapentine regimen is one of the earliest reported after the controlled clinical trials (6). The regimen shows promise for congregate settings, where treatment is convenient for the patients and efficient for the health department. Adverse events in this report resembled those in the treatment trials and limited or changed treatment for three of 65 patients. CDC is collaborating with health departments and institutions nationwide in collecting data on this regimen in routine usage.

What is already known on this topic?

Tuberculosis (TB), caused by a contagious airborne bacterium, can be widely transmitted in congregate settings. Tracing contacts and treating new infections are complex, time-intensive, interventions in congregate settings, and completion of treatment for preventing TB is historically 70% or less. An investigative approach starting with contacts who had the most exposure, with interim analyses of findings, clarifies the need for including contacts with less exposure. In jurisdictions with low TB incidence, TB control programs might not have sufficient local resources to respond to extensive transmission.

What is added by this report?

Screening at a school of 1,249 (90.4%) contacts of a student with TB found one person with pulmonary TB disease and 162 with latent *Mycobacterium tuberculosis* infection (LTBI), of whom 159 started LTBI treatment regimens for preventing progression to TB disease and 153 completed a regimen. A state emergency response plan pulled together dozens of health professionals, who devoted hundreds of hours to testing persons who were exposed to TB and providing care for those with TB disease and LTBI.

What are the implications for public health practice?

TB control programs and other public health agencies should be aware that investigating TB in a school can outstrip the response capabilities of local agencies and require large-scale mobilization with state and county leadership. Public health agencies should have a plan for keeping the public informed and educated about TB and apportion the necessary resources to meet the acute needs until they are resolved.

Of contacts initially diagnosed with LTBI in this investigation, 99% started treatment, which exceeded the 2010 U.S. treatment-start rate of 72% (Division of Tuberculosis Elimination, National Center for HIV/AIDS, Viral Hepatitis, STD, and TB Prevention, CDC, unpublished data, 2010). The two short-course regimens including either rifampin or rifapentine for LTBI treatment probably contributed to the treatment completion rate of 98% of those starting an LTBI-specific regimen. For Colorado overall, the treatment completion rate for infected contacts who started self-supervised daily isoniazid in 2007–2011 was 73% (Colorado Department of Public Health and Environment, unpublished data, 2013), although in this report, all three contacts who started this regimen completed it. For the United States overall, the completion rate in 2010 was 68%. The campaign for public education in Colorado might have facilitated the successes at the school.

Drug-susceptible TB disease is curable, but its historical reputation as a lethal contagious disease generates stigma, and misinformation can amplify fears. When communicating to the public about a crisis, the information should be simple, credible, accurate, consistent, and on time. One of the best ways to counter the public’s fears is to provide useful information about the event and let them know what they can do (7).

## Figures and Tables

**TABLE:** Rates of *Mycobacterium tuberculosis* infection among groups of school contacts* of a student with tuberculosis (TB) disease, by decreasing durations of exposure — Colorado, 2011–2012

Group†	Sought	Medically evaluated§	Initial diagnosis		Final diagnosis		Infection rate (%)¶
	
			TB disease	LTBI	TB disease	LTBI	
Students who had two or more classes with the index patient and teachers**	19	19	0	10	0	10	53
Students who had one class with the index patient	140	140	1	49	0	50	36
All others	1,222	1,090	1	102	1	102	9
**Overall school contacts**	**1,381**	**1,249**	**2**	**161**	**1**	**162**	**13**

**Abbreviation:** LTBI = latent *M. tuberculosis* infection.

* Does not include five household contacts who were evaluated before the investigation at the school and 20 nonschool social contacts who were sought after the investigation.

† Mutually exclusive contact groups, listed by decreasing relative durations of exposure.

§ Fully medically evaluated for LTBI or TB disease; interviewed for TB symptoms and tested individually as medically indicated.

¶ Total infections (TB disease and LTBI) for the group; denominator is the number of contacts fully medically evaluated.

** The six teachers of the index patient, regardless of number of classes; other teachers and school personnel are grouped with “All others.”
